# Driving Performance and Cannabis Users’ Perception of Safety: A Randomized Clinical Trial

**DOI:** 10.1001/jamapsychiatry.2021.4037

**Published:** 2022-01-26

**Authors:** Thomas D. Marcotte, Anya Umlauf, David J. Grelotti, Emily G. Sones, Philip M. Sobolesky, Breland E. Smith, Melissa A. Hoffman, Jacqueline A. Hubbard, Joan Severson, Marilyn A. Huestis, Igor Grant, Robert L. Fitzgerald

**Affiliations:** 1 Department of Psychiatry, University of California San Diego, San Diego; 2 Department of Pathology, University of California San Diego, San Diego; 3 Department of Pathology and Laboratory Medicine, Santa Clara Valley Medical Center, San Jose, California; 4 LetsGetChecked Labs, Monrovia, California; 5 Vividion Therapeutics, San Diego, California; 6 Department of Pathology and Laboratory Medicine, Dartmouth-Hitchcock Medical Center, Lebanon, New Hampshire; 7 Brainbaseline, Iowa City, Iowa; 8 Institute for Emerging Health Professions, Thomas Jefferson University, Philadelphia, Pennsylvania

## Abstract

**Importance:**

Expanding cannabis medicalization and legalization increases the urgency to understand the factors associated with acute driving impairment.

**Objective:**

To determine, in a large sample of regular cannabis users, the magnitude and time course of driving impairment produced by smoked cannabis of different Δ9-tetrahydrocannabinol (THC) content, the effects of use history, and concordance between perceived impairment and observed performance.

**Design, Setting, and Participants:**

This double-blind, placebo-controlled parallel randomized clinical trial took place from February 2017 to June 2019 at the Center for Medicinal Cannabis Research, University of California San Diego. Cannabis users were recruited for this study, and analysis took place between April 2020 and September 2021.

**Interventions:**

Placebo or 5.9% or 13.4% THC cannabis smoked ad libitum.

**Main Outcomes and Measures:**

The primary end point was the Composite Drive Score (CDS), which comprised key driving simulator variables, assessed prior to smoking and at multiple time points postsmoking. Additional measures included self-perceptions of driving impairment and cannabis use history.

**Results:**

Of 191 cannabis users, 118 (61.8%) were male, the mean (SD) age was 29.9 (8.3) years, and the mean (SD) days of use in the past month was 16.7 (9.8). Participants were randomized to the placebo group (63 [33.0%]), 5.9% THC (66 [34.6%]), and 13.4% THC (62 [32.5%]). Compared with placebo, the THC group significantly declined on the Composite Drive Score at 30 minutes (Cohen *d* = 0.59 [95% CI, 0.28-0.90]; *P* < .001) and 1 hour 30 minutes (Cohen *d* = 0.55 [95% CI, 0.24-0.86]; *P* < .001), with borderline differences at 3 hours 30 minutes (Cohen *d* = 0.29 [95% CI, –0.02 to 0.60]; *P* = .07) and no differences at 4 hours 30 minutes (Cohen *d* = –0.03 [95% CI, –0.33 to 0.28]; *P* = .87). The Composite Drive Score did not differ based on THC content (likelihood ratio χ^2^_4_ = 3.83; *P* = .43) or use intensity (quantity × frequency) in the past 6 months (likelihood ratio χ^2^_4_ = 1.41; *P* = .49), despite postsmoking blood THC concentrations being higher in those with the highest use intensity. Although there was hesitancy to drive immediately postsmoking, increasing numbers (81 [68.6%]) of participants reported readiness to drive at 1 hour 30 minutes despite performance not improving from initial postsmoking levels.

**Conclusions and Relevance:**

Smoking cannabis ad libitum by regular users resulted in simulated driving decrements. However, when experienced users control their own intake, driving impairment cannot be inferred based on THC content of the cigarette, behavioral tolerance, or THC blood concentrations. Participants’ increasing willingness to drive at 1 hour 30 minutes may indicate a false sense of driving safety. Worse driving performance is evident for several hours postsmoking in many users but appears to resolve by 4 hours 30 minutes in most individuals. Further research is needed on the impact of individual biologic differences, cannabis use history, and administration methods on driving performance.

**Trial Registration:**

ClinicalTrials.gov Identifier: NCT02849587

## Key Points

**Question:**

What factors are related to the impact of smoked cannabis on driving and the users’ perception of driving ability?

**Findings:**

In this randomized clinical trial of 191 regular cannabis users who smoked ad libitum placebo or 5.9% or 13.4% Δ9-tetrahydrocannabinol (THC) cigarettes, simulator driving worsened in the THC group, but this was unrelated to THC content, use history, or blood THC concentration. Perception of driving impairment decreased at 1 hour 30 minutes, despite no objective improvement in driving; on average, performance was indistinguishable from the placebo group at 4.5 hours.

**Meaning:**

When experienced cannabis users control their own intake, one cannot infer impairment based on the product THC content or blood concentrations, and the disconnect between performance and self-perceived impairment is an important public safety message.

## Teaser

This randomized clinical trial studies the magnitude and time course of effects of cannabis on driving, effect of cannabis with different tetrahydrocannabinol amounts, possible tolerance effects, and accuracy of self-perception of impairment.

## Introduction

As jurisdictions legalize cannabis for medicinal and recreational use, there are growing concerns regarding a potential increased prevalence of cannabis-impaired drivers.^1,2^ Acute consumption of Δ9-tetrahydrocannabinol (THC) negatively affects cognitive functioning^3^ and reduces driving performance, particularly in lane position control (standard deviation of lateral position)^4,5,6,7,8,9^ and ability to adjust to lead car speed changes (car following^10^). However, epidemiologic data regarding the effect of legalization on crash risk are not consisent.^11,12,13,14^ The varied findings partially reflect challenges in accessing robust prelegalization and postlegalization data and determining acute intoxication^1^ but also show a disconnect between impairing effects observed in controlled studies and expectations regarding crash rates.

Questions remain regarding the magnitude and time course of the effects of cannabis on those most likely to be on the road (regular users smoking to a desired level of intoxication) as well as the effect of different product THC amounts. While seminal studies examined these questions, most used small sample sizes (eg, <25 participants), low–THC content product within a crossover design, and structured dosing protocols, with some exceptions, for example using an ad libitum approach.^5^ Such studies provide critical data regarding THC dose effects but do not reflect real-world use. This is particularly important given concerns that the increasing THC content of products may result in greater impairment. Small sample sizes may also limit generalizability, while crossover designs using psychoactive substances present blinding challenges.^15^

The appropriate waiting period before driving after cannabis smoking is also a significant public safety concern, with some suggesting 3 to 5 hours^16,17,18^ and others recommending longer.^19^ Because this decision may be self-determined based on feeling impaired, it is important to understand the accuracy of these self-evaluations. In addition, while frequency of cannabis use is associated with increased behavioral tolerance,^20^ the relationship to driving remains poorly understood because individuals may counteract tolerance by consuming greater amounts to achieve desired psychoactive effects. Recent systematic reviews concluded that major limitations in cannabis-related driving research include a lack of studies examining regular users over a 4- to 6-hour postsmoking time frame^21^ as well as small sample sizes.^22^

Within a sample of nearly 200 regular cannabis users instructed to smoke cannabis as they do at home to achieve a usual level of intoxication, the aims of this study were to determine, with respect to driving outcomes, the (1) magnitude and time course of effects, (2) effect of cannabis with different THC amounts, (3) possible tolerance effects, and (4) accuracy of self-perception of impairment.

## Methods

The study was approved by the Human Research Protections Program at the University of California, San Diego; the US Food and Drug Administration; and the Research Advisory Panel of California. The study was conducted in accordance with the Declaration of Helsinki.^23^ Consolidated Standards of Reporting Trials (CONSORT) reporting guideline were followed.

### Participants

Participants were recruited in San Diego, California, via fliers, community outreach, and ClinicalTrials.gov from February 2017 to June 2019. Inclusion criteria were age 21 to 55 years, using cannabis 4 or more times in the past month, holding a valid driver’s license, driving at least 1000 miles in the past year, and willing to abstain from cannabis for 2 days prior to the training and experimental study days.

Exclusion criteria were history of traumatic brain injury; significant cardiovascular, hepatic, or kidney disease; uncontrolled hypertension; chronic pulmonary disease; positive pregnancy test; positive urine screen for cocaine, amphetamines, opiates, and phencyclidine; current (past-year) substance use disorder (no participant met criteria for cannabis use disorder); history of schizophrenia, bipolar depression with mania, and/or current suicidal ideation; unwilling to refrain from driving after consuming study medication; and oral fluid THC more than >5 ng/mL on the testing day. Participants provided written informed consent. Data on race and ethnicity were self-reported.

### Study Design

The trial protocol is available in Supplement 1. This was a double-blind, placebo-controlled, parallel clinical trial in which participants were randomized using permuted blocks stratified by prior cannabis exposure (using ≥4 times per week or <4 times per week in the past month, based on stratifications that previously differentiated among users^9,24^) to smoke a cannabis cigarette with either 13.4%, 5.9%, or 0.02% THC (placebo) content. Participants were instructed to abstain from cannabis for 48 hours prior to the training and experimental days and underwent a 1-hour simulator training session prior to the testing day. The training session exposed participants to all of the individual components of the drive, culminating in a 25-minute drive similar to what they would encounter on the testing day. On the experimental day, they completed a urine drug screen and breathalyzer for alcohol and drugs and oral fluid sample for THC presence (Draeger 5000). If the oral fluid was positive (>5-ng/mL THC), suggesting relatively recent use, the assessment was canceled. Oral fluid samples were also quantified by liquid chromatography/tandem mass spectrometry as the final indicator of possible recent use, with participants having oral fluid with more than 5-ng/mL THC excluded from analyses. Participants completed driving simulations and blood collections prior to and following cannabis smoking (detailed toxicology findings reported elsewhere^25,26^). The primary outcome was the Composite Drive Score (CDS), a measure composed of key driving simulator variables.

### Driving Simulations

Driving simulations, approximately 25 minutes in length, were presented on a STISIM M300WS-Console Driving Simulator System (Systems Technology, Inc) consisting of 3-screen, wide field-of-view monitors, steering wheel, and accelerator and brake pedals, and programmed using STISIM Drive version 3.14.^27^ The simulations emulated city and country driving, including common traffic challenges (eg, freeway merging), as well as scenarios providing outcomes similar to those widely used in drug-impaired driving studies.^5,7,8,28^ At a specified distance, participants completed a modification of the Surrogate Reference Task,^29^ which required participants to maintain their lane position and speed in a straight roadway, while responding to a divided attention task on an iPad to the side of the dashboard. Key variables included standard deviation of lateral position or swerving, standard deviation (variability) of speed, and number of correct divided attention stimuli identified while driving. At another distance, car following required participants to adjust their speed to a lead car that speeds up and slows down according to a sinusoidal wave. The key variable is coherence between the participant and lead car (a correlation ranging from 0-1). CDS, comprising the key variables described above, normalized to a common metric (*z* scores derived from the presmoking drive of all participants), was calculated to globally represent driving performance and, by not being dependent on a single outcome variable, provided a more stable indicator of driving performance (eAppendix in Supplement 2). A higher score indicated worse performance. Similar approaches have been used elsewhere^30,31^ and address concerns regarding the use of multiple dependent outcomes in cannabis and driving research.^22^ Postsmoking driving simulations occurred approximately 30 minutes, 1 hour 30 minutes, 3 hours 30 minutes, and 4 hours 30 minutes after smoking.

### Study Drug and Administration

Bulk cannabis plant material containing 5.9% THC, 13.4% THC, or placebo was acquired from the National Institute on Drug Abuse Drug Supply Program and hand rolled into 700-mg cigarettes. An ad libitum regimen was used within a negative pressure room, with participants instructed to “smoke the cigarette the way you do at home to get high. You may take up to 10 minutes.” A minimum of 4 puffs was required. Venous blood was collected from an indwelling intravenous arm catheter (eAppendix in Supplement 2).

### Perceptions of Impairment

After smoking, but prior to each driving session, participants were asked “how high are you?”, “how impaired are you to drive?” (both ratings from 0 [not at all] to 100 [extremely]), and “would you drive in your current state?” (yes/no). After each postsmoking driving session, participants were asked “how much did the study drug affect your driving?” (0 [not at all] to 100 [extremely]) as well as “how well did you drive?” (0 [not at all well] to 100 [extremely well]).

### Statistical Analysis

Generalized least squares models were used for numeric outcomes with covariance structure selected by minimum Akaike information criterion. Poisson and logistic regression models with generalized estimating equation method were used for discrete and binary outcomes, respectively. Time was treated as a factor to accommodate nonlinear changes in the outcomes. Treatment was first considered as a 3-level variable (placebo, 5.9% THC, and 13.4% THC) and then as a 2-level treatment variable (placebo and THC) where the 5.9% and 13.4% groups were combined. For all models, 3 terms were included: treatment, time (5 time points), and treatment-time interaction. For effect sizes estimating differences at multiple time points, correction for multiple comparisons was applied using false discovery rate method (subscore and secondary analyses only).

Cannabis use intensity, estimated as total THC exposure, was based on self-reported frequency and quantity of use in the past 6 months using a timeline follow-back approach and split into 3 groups (lowest quartile, 2 middle quartiles combined, and highest quartile; eAppendix in Supplement 2). Two-sided *P* values were statistically significant at .05. Analysis were conducted between April 2020 and September 2021.

## Results

**Figure 1.**
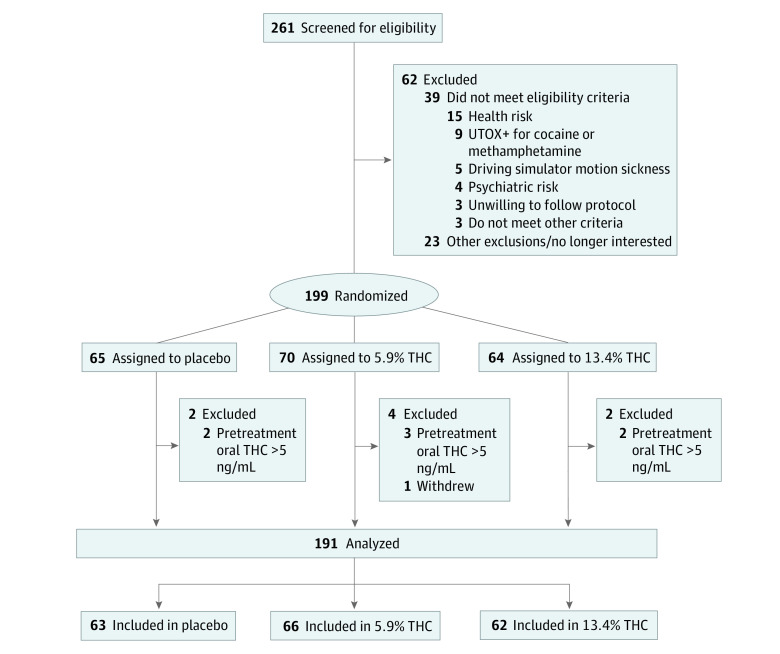
CONSORT Diagram Showing Participant Inclusion/Exclusion From Initial Screening to Final Sample UTOX+ indicates urine toxicology; THC, tetrahydrocannabinol.

Of 261 individuals screened for eligibility, 199 were randomized to 1 of 3 arms: placebo (63 [33.0%]), 5.9% THC (66 [34.6%]), or 13.4% THC (62 [32.5%]) (Figure 1). Seven were subsequently excluded owing to presmoking elevated oral fluid THC levels and 1 withdrew immediately postsmoking. The final sample was 191 participants (118 men [61.8%]; mean [SD] age, 29.9 [8.3] years) who used cannabis a mean (SD) of 16.7 (9.8) days in the past 30 days, approximately 1 cigarette (0.5 g) when using, with 98 (51.3%) using less than 4 times per week. There were no significant group differences on key background variables (Table 1).

**Table 1.** Demographic Characteristics of Study Participants by Treatment Group

Characteristic	Placebo (n = 63)	5.9% THC (n = 66)	13.4% THC (n = 62)	*P* value
Age, mean (SD), y	28.1 (7.3)	30.7 (8.8)	30.9 (8.6)	.11
Male, No. (%)	32 (50.8)	47 (71.2)	39 (62.9)	.057
Female, No. (%)	31 (49.2)	19 (28.8)	23 (37.1)	
Education, mean (SD), y	15.0 (1.9)	14.9 (2.0)	15.3 (2.0)	.44
Race and ethnicity, No. (%)				
African American	8 (12.7)	6 (9.1)	4 (6.5)	.62
Asian	5 (7.9)	8 (12.1)	4 (6.5)	
Hispanic	15 (23.8)	19 (28.8)	22 (35.5)	
Indigenous	5 (7.9)	2 (3.0)	1 (1.6)	
Multiracial	2 (3.2)	3 (4.5)	2 (3.2)	
Non-Hispanic White	28 (44.4)	28 (42.4)	27 (43.5)	
Unknown	0	0	2 (3.2)	
Miles driven past year, median (IQR)	8730 (5420-12 825)	9300 (5298-12 665)	8280 (5040-13 320)	.97
Cannabis				
Current cannabis use <4 times/wk, No. (%)	34 (54.0)	33 (50.0)	31 (50.0)	.88
Days used, mean (SD), last 30 d	16.9 (9.7)	16.0 (9.6)	17.3 (10.2)	.77
Grams/d when using, median (IQR), last 30 d	0.55 (0.25-1)	0.55 (0.30-1)	0.50 (0.25-1)	.62

Abbreviation: THC, tetrahydrocannabinol.

### Smoking Topography and Blinding

There were no significant group differences in grams of cannabis/placebo material used during the session (estimated from the weight returned) (mean [SD]: placebo, 0.47 [0.17]; 5.9% THC, 0.44 [0.17], 13.4% THC, 0.43 [0.15]; *P* = .42). At approximately 15 minutes after smoking initiation, there was a significant difference (*P* < .001) in blood THC concentrations between all 3 groups (mean [SD]: placebo, 1.3 [1.9] ng/mL; 5.9 THC%, 50.6 [40.8] ng/mL; 13.4% THC, 32.7 [29.3] ng/mL), with the 5.9% THC group reaching the highest concentration.^32^ A total of 117 individuals (92%) in the THC group correctly guessed their treatment assignment. There was no difference between the 5.9% THC and 13.4% THC groups (62 [94%] vs 55 [90%]; *P* = .61]); 30 (48.3%) in the placebo group believed they received active THC.

### Primary Outcomes

#### Crashes

There were no significant differences between the 3 groups on the number of crashes at any time point (odds ratio range, 0.78-1.57; *P* > .75).

#### CDS

**Figure 2.**
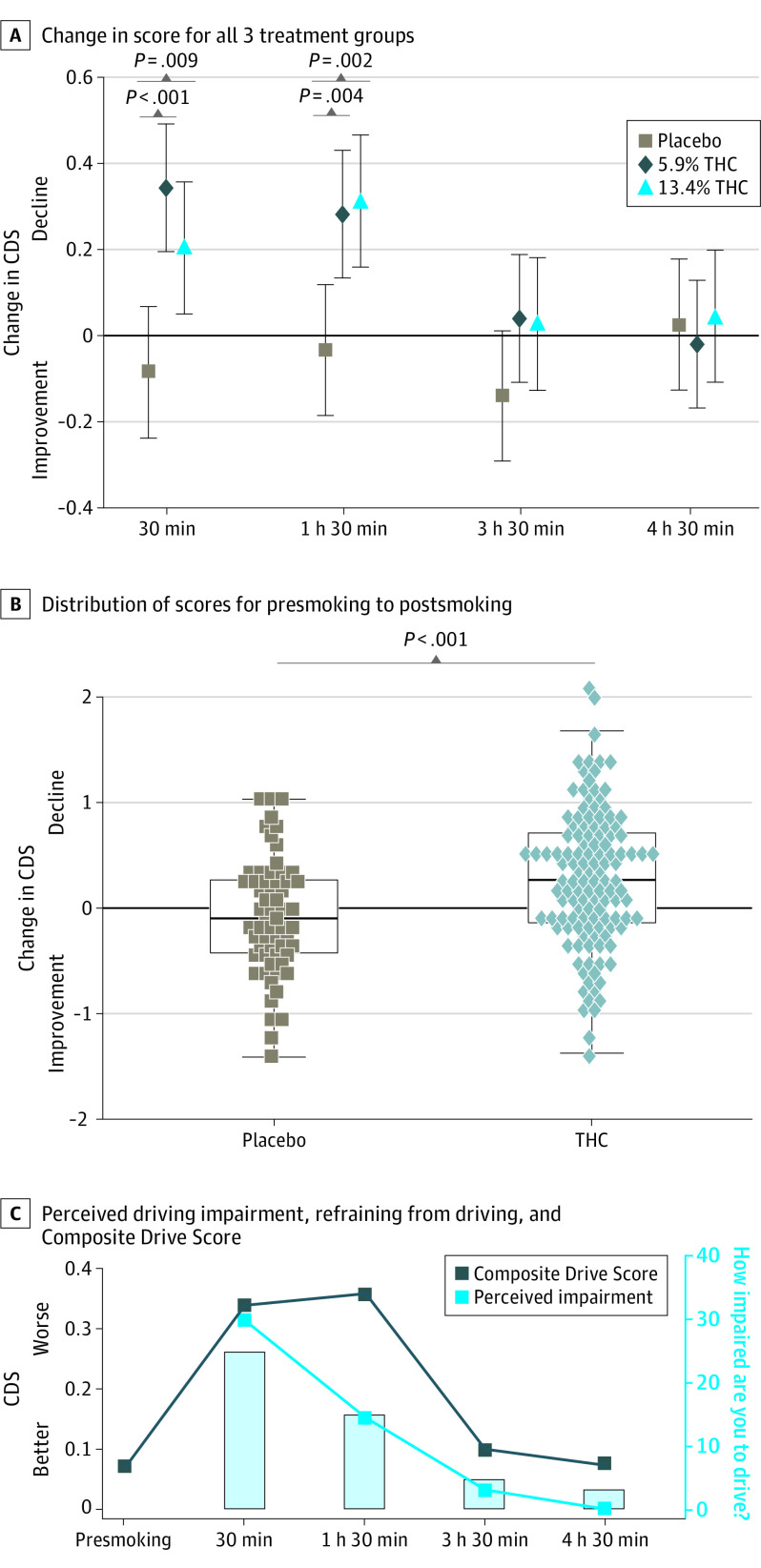
Change in Simulator Performance Over Time, Distribution of Simulator Changes at 30 Minutes, and Relationship Between Self-reported Impairment and Objective Driving Simulator Performance A, Change in Composite Drive Score (CDS) from baseline: all 3 treatment groups. Values are means (95% CIs). B, Distribution of changes in the CDS from presmoking to 30 minutes postsmoking; shapes represent individual values, the boxes show the 25th, 50th (median), and 75th percentiles. C, Relationship between participant median self-report of driving impairment (light blue line), willingness to refrain from driving (columns showing percent of participants who would not drive: 30 minutes, 52.5%; 1 hour 30 minutes, 31.4%; 3 hours 30 mininutes, 10.1%; and 4 hours 30 minutes, 6.8%), and CDS (dark blue line) (tetrahydrocannabinol [THC] participants only).

Compared with placebo, the THC groups had a significant decline in CDS performance; there were no differences between the 2 THC groups in change over time (likelihood ratio χ^2^_4_ *=* 3.83; *P* = .43; Figure 2A). Thus, the 2 groups were combined for subsequent analyses (eFigure in Supplement 2).

Table 2 summarizes the CDS results, with change from presmoking score as the primary outcome. Compared with changes in the placebo group, the THC group had significantly greater declines at 30 minutes and 1 hour 30 minutes. The differences were no longer statistically significant at 3 hours 30 minutes (Cohen *d* = 0.29 [95% CI, –0.02 to 0.60]; *P* = .07) or 4 hours 30 minutes (Cohen *d* = –0.03 [95% CI, –0.33 to 0.28]; *P* = .87). The CDS did not differ by sex (Cohen *d* = 0.18 [95% CI, –0.04 to 0.41]; *P* = .11) and controlling for sex (because THC/placebo groups differed: 86 [67.2%] vs 32 [50.8%] were male, respectively; *P* = .049) did not change results. There were no significant practice effects in the placebo group (Table 2).

**Table 2.** Composite Drive Score for the Placebo and THC Groups at Each Time Point

Time point	Composite Drive Score		Change in mean Composite Drive Score from time 1^a^					
	Mean (SD)		Placebo^b^		THC^c^		Difference (THC vs placebo)^d^	
	Placebo group	THC group	Cohen *d* (95% CI)	*P* value	Cohen *d* (95% CI)	*P* value	*C*ohen *d (*95% CI)	*P* value
1 (Presmoke)	–0.09 (0.64)	0.06 (0.55)	NA	NA	NA	NA	NA	NA
2 (30 min)	–0.17 (0.61)	0.34 (0.61)	–0.14 (–0.39 to 0.11)	.27	0.45 (0.28 to 0.63)	<.001	0.59 (0.28 to 0.90)	<.001
3 (1 h 30 min)	–0.13 (0.61)	0.36 (0.62)	–0.06 (–0.31 to 0.19)	.64	0.49 (0.31 to 0.67)	<.001	0.55 (0.24 to 0.86)	<.001
4 (3 h 30 min)	–0.23 (0.59)	0.10 (0.61)	–0.24 (–0.49 to 0.02)	.07	0.05 (–0.13 to 0.23)	.56	0.29 (–0.02 to 0.60)	.07
5 (4 h 30 min)	–0.07 (0.66)	0.07 (0.57)	0.04 (–0.21 to 0.29)	.76	0.01 (–0.16 to 0.19)	.88	–0.03 (–0.33 to 0.28)	.87

Abbreviations: NA, not applicable; THC, tetrahydrocannabinol.

^a^ The test for the overall significance in differences of changes between the THC and the placebo was statistically significant (*P* < .001).

^b^ Each placebo time point score compared with the placebo presmoking score.

^c^ Each THC time point score compared with the THC presmoking score.

^d^ Comparison of change from baseline between placebo and THC groups.

The THC group performed significantly worse than the placebo group at 30 minutes, although some participants performed similarly to those in the placebo group (Figure 2B). Based on a 15th percentile cut point in the distribution of CDS change scores from the placebo group, 57 of 125 individuals (45.6%) in the THC group would be classified as impaired at 30 minutes (eAppendix in Supplement 2).

#### Drive Subscores

The changes in performance for the individual driving variables comprising the CDS (collected at the specified distances) were generally consistent with the CDS, showing significant changes at 30 minutes and at 1 hour 30 minutes (eTable 1 in Supplement 2). Differences in changes on the divided attention task were only seen at 30 minutes. In addition, time driving out of lane during the modified Surrogate Reference Task was significantly different at 1 hour 30 minutes.

### Perception of Effects and Performance

After smoking, but prior to driving, the THC group reported being significantly more impaired to drive at all time points, with the rating dropping at each time point (eTable 2A in Supplement 2). At 30 minutes, 57 of 120 (47.5%) in the THC group would drive in their current state; this number increased to 81 (68.6%) at 1 hour 30 minutes, 107 (90%) at 3 hours 30 minutes, and 110 (93.2%) at 4 hours 30 minutes (eTable 2B in Supplement 2; Figure 2C).

After driving, the THC group rated cannabis as affecting their performance more than the placebo group at all time points. However, their rating of how well they drove was worse than the placebo group only at 30 minutes (eTables 3A and B in Supplement 2).

### Driving Performance and THC Blood Concentrations

**Figure 3.**
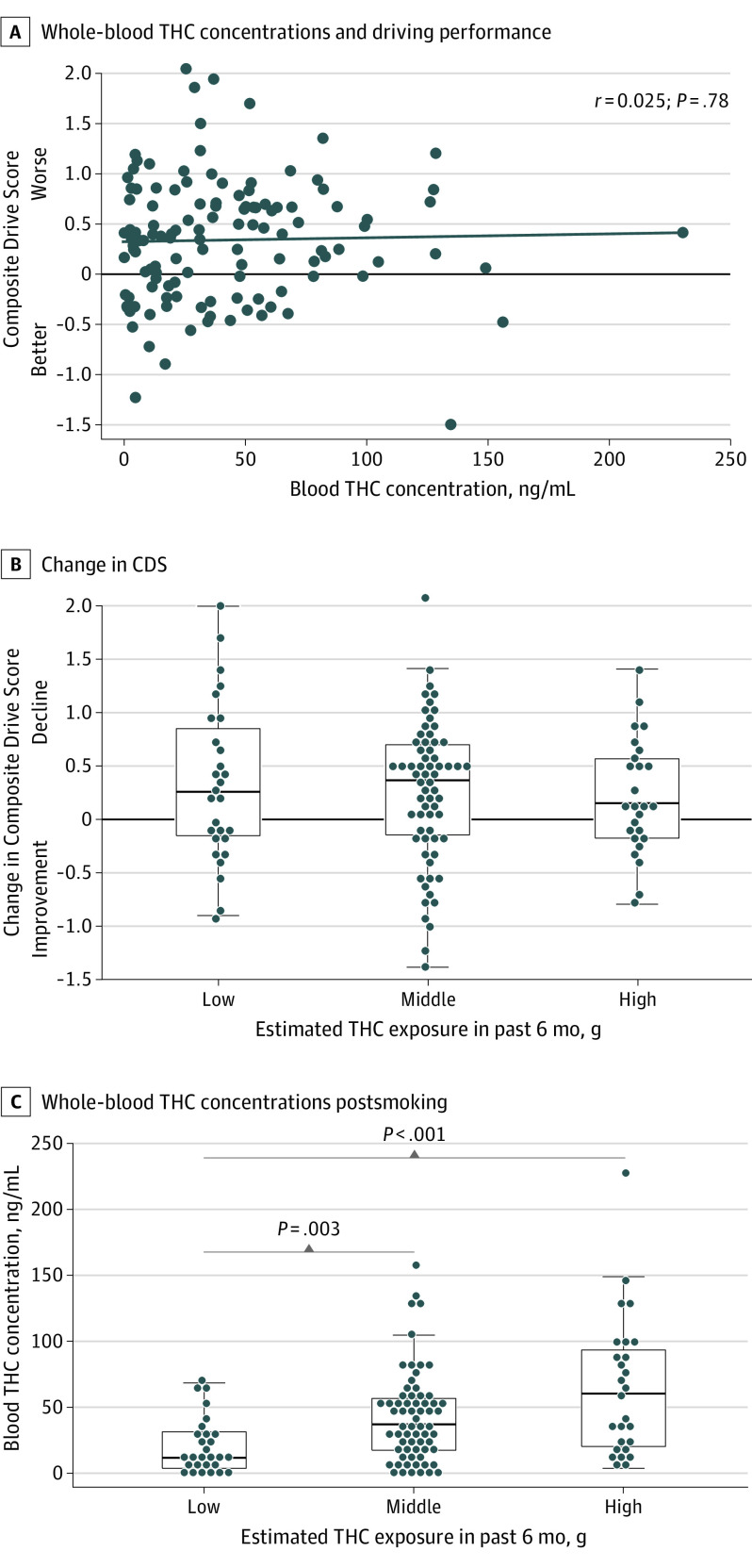
Relationship Between Initial Postsmoking Driving Performance and Whole Blood THC Concentrations and Between Use Intensity Over the Past 6 Months and Composite Drive Score at 30 Minutes and Whole-Blood THC Concentrations Immediately Postsmoking A, Relationship between whole-blood tetrahydrocannabinol (THC) concentrations and driving performance at 30 minutes postsmoking (THC group only). B, Change in Composite Drive Score based on use history in the past 6 months at 30-minute time point (THC group only). C, Whole-blood THC concentrations immediately postsmoking, based on use history in the past 6 months (THC group only).

Within the THC group, there was no relationship between blood THC concentrations at 30 minutes and the CDS (*r =* .025, *P* = .78; Figure 3A) or any of the subsequent time points (eTable 4 in Supplement 2).

### Cannabis Use History (Intensity) and Driving Performance

Within the THC group, after smoking there were no differences between the subgroups with the highest, middle, or lowest intensity of use (in the past 6 months) in how high they felt (*F*_2,112_ = 1.75; *P* = .18), nor the CDS changes at 30 minutes (*F*_2,117_ = 0.243; *P* = .79; Figure 3B) or across all time periods (*F*_8,581_ = 1.08; *P* = .38). However, postsmoking blood THC concentrations significantly differed across all 3 groups (Kruskal-Wallis χ^2^_2_ = 16.3; *P* < .001), with the lowest-intensity group having the lowest concentrations (median [IQR], 11.8 [3.8-31.4] ng/mL; *P* = .003 vs middle, *P* < .001 vs high) and the highest-intensity group having the highest concentrations (median [IQR], 60.5 [20.4-93.5] ng/mL; *P* = .08; middle: median [IQR], 37.2 [17.5-56.4] ng/mL) (Figure 3C).

## Discussion

In this study of 191 regular cannabis users randomized to smoke THC or placebo cigarettes ad libitum, we found worse performance in the THC group on a measure of overall driving simulator performance as well as specific driving challenges, including a divided attention task, adding to a growing literature that THC negatively impacts driving ability.^5,33,34^ The magnitude of the effect was in the medium range (Cohen *d* of approximately 0.50^35^), suggesting a nontrivial difference.

When instructed to “smoke as you would at home to get high,” we found no significant differences in driving performance or THC blood concentrations,^32^ based on the THC content of the cannabis, supporting the importance of smoking topography (deepness of inhalation, period of holding, etc).^5,36,37,38^ There is concern that the increasing THC content in products will result in significantly greater road safety risks. However, the current study suggests that some users may smoke such products in a manner that results in no greater impairment than lower-THC products. These findings do not necessarily translate to other methods of administration, such as dabbing, vaping, and oral consumption where self-titration is more difficult, although a recent study suggests concentrate users may self-titrate.^39^

While the THC group generally reported feeling impaired and hesitant to drive at 30 minutes, at 1 hour 30 minutes participants increasingly rated themselves as safe to drive, whereas simulator data indicated ongoing reduced driving performance (Figure 2C), including being more likely to leave their lane. These first few hours may constitute a period of greatest risk because users who are self-evaluating whether it is safe to drive may be less likely to refrain from driving or to attempt to compensate for reduced functioning. This is an important topic for public safety messaging, since a goal is to keep impaired drivers off of the road prior to becoming a danger.

The effect size seen at 3.5 hours (Cohen *d* = 0.29) suggests lingering impairment in some participants, although the THC group’s driving was no longer statistically different from controls (*P* = .07). THC-associated driving reductions were resolved by 4.5 hours in most participants. This is generally consistent with the time frame noted in studies using lower–THC content materials.^5,6,7,40,41,42^ It is possible that impairments in other, unmeasured abilities may persist^43^ or become apparent over longer drives, although a recent 60-minute on-road study concluded that no negative THC effects were seen 4 to 5 hours after use.^6^

There was no correlation between blood THC concentrations collected 15 minutes after smoking and simulator performance at 30 minutes or any other time point even under our highly controlled conditions. In the real world, the time from consumption to a law enforcement stop and subsequent blood collections is highly variable, and the current results reinforce that per se laws based on blood THC concentrations are not supported.^34,44^

Greater intensity of cannabis use in the past 6 months was associated with reaching higher blood THC concentrations following smoking but not self-reported greater levels of highness nor worse driving performance than lower-intensity groups, consistent with development of behavioral tolerance.^20^ However, the current findings also suggest that when instructed to achieve a self-determined level of highness, users with a history of greater use intensity adapted to tolerance by increasing THC exposure, resulting in performance decrements similar to users with lower-intensity use and that they may not be less of a driving risk. Behavioral tolerance benefits may be more apparent in medicinal users who target specific symptoms (eg, pain) and maintain a consistent dosing level.

Lastly, based on the distribution of the placebo group, approximately half of the THC group would be categorized as impaired, suggesting that identifying those at greatest risk for impairment is not as straightforward as detecting recent use and remains an important public safety challenge. It is worth noting that alcohol exhibits a more consistent linear effect between blood (alcohol) levels and driving impairment, although even in that case there is significant variability between studies (and individuals) in the relationship between levels of ingestion and reductions in driving performance.^45^

### Limitations

This study has a number of limitations. With the aim of maximizing ecological validity, we had individuals smoke to the level of highness they desire and thus did not address participants reaching particularly elevated highness levels, nor the effects of controlled dosing. However, it should be noted that studies using controlled smoking methods also find substantial variability in blood THC concentrations, suggesting an influence of smoking topography.^46^ The study may not be generalizable to infrequent or naive users, vulnerable populations (eg, older persons, individuals with medical conditions), or other routes of administration for which self-titration is difficult (eg, edibles) and thus may underestimate the effects of THC on driving in the broader, general public. Because the study did not include a nonuser control group, the study only addresses how regular users exposed to THC perform on the CDS compared with regular users receiving placebo. There is evidence that acute cannabis use can impair visual function (and driving); we cannot determine the specific correlates of reduced driving (cognitive, visual) because these were not comprehensively assessed.^30^ Classification of individuals as impaired on experimental driving simulator scenarios is dependent on the size/composition of the reference group and may differ with other samples. Because no measurements were made between 1 hour 30 minutes and 3 hours 30 minutes, we cannot comment on the timing of the maximum decline in driving score, nor the recovery trajectory during his period. The potential cumulative effects of serial smoking were not addressed. Lastly, while the simulations captured a reasonable sampling of driving behavior, we were unable to address whether performance over longer driving periods might show impairment.

## Conclusions

In a placebo-controlled parallel study of regular cannabis users smoking cannabis with different THC content ad libitum, there was statistically significant worsening on driving simulator performance in the THC group compared with the placebo group. The THC content of the cannabis and intensity of prior cannabis use were not associated with driving outcomes; participants self-titrated in a manner that yielded similar reductions in driving performance, despite achieving different THC blood concentrations. A lack of insight regarding driving impairments, particularly at 90 minutes, is of concern, given that users will likely self-evaluate when they feel safe to drive. Although performance was improving at 3.5 hours, recovery was not fully seen until 4.5 hours postsmoking. The fact that not all participants consuming THC met the criteria for impairment underscores the interindividual variability seen with the impairing effects of cannabis.^47^ The lack of relationship between blood THC concentration and driving performance raises questions about the validity of per se laws. When users control their own intake, one cannot infer the level of impairment based on the THC content of the product, the level of behavioral tolerance in the individual, or the blood THC concentration. Future research should address factors such as individual biologic differences, personal experience with cannabis, and cannabis administration methods in relation to driving impairment.

## Notes

Supplement 1. Trial Protocol

Supplement 2. eTable 1. Drive Subscores eTable 2. Participant responses to self-report questionnaire after smoking, but prior to driving eTable 3. Driving simulator self-assessment questionnaire by group for each time point after driving eTable 4. Correlation between blood THC levels and the Composite Driving Score at each timepoint eFigure. Change in Composite Drive Score from baseline: THC groups combined eAppendix eReferences

Supplement 3. Data Sharing Statement
