# Supplementary material for: Driving Performance and Cannabis Users’ Perception of Safety: A Randomized Clinical Trial
Source: JAMA Psychiatry. 2022 Jan 26;79(3):1–9. doi: 10.1001/jamapsychiatry.2021.4037 (PMC8792796; doi:10.1001/jamapsychiatry.2021.4037)
Supplement: Supplement 2. — eTable 1. Drive Subscores eTable 2. Participant responses to self-report questionnaire after smoking, but prior to driving eTable 3. Driving simulator self-assessment questionnaire by group for each time point after driving eTable 4. Correlation between blood THC levels and the Composite Driving Score at each timepoint eFigure. Change in Composite Drive Score from baseline: THC groups combined eAppendix eReferences [file jamapsychiatry-e214037-s002.pdf]

Supplemental Online Content

Marcotte TD, Umlauf A, Grelotti DJ, et al. Driving performance and cannabis users' perception of safety: a randomized clinical trial. *JAMA Psychiatry*. Published online January 26, 2022. doi:10.1001/jamapsychiatry.2021.4037

eTable 1. Drive Subscores
eTable 2. Participant responses to self-report questionnaire after smoking, but prior to driving
eTable 3. Driving simulator self-assessment questionnaire by group for each time point after driving
eTable 4. Correlation between blood THC levels and the Composite Driving Score at each timepoint
eFigure. Change in Composite Drive Score from baseline: THC groups combined
eAppendix
eReferences

This supplemental material has been provided by the authors to give readers additional information about their work.

© 2022 Marcotte TD et al. *JAMA Psychiatry.*

eTable 1. Drive Subscores

		Time 1 (pre-smoke)	*Time 2 (30min)*	*Time 3 (1h 30min)*	*Time 4 (3h 30min)*	*Time 5 (4h 30min)*
**DRIVING VARIABLES DURING mSuRT**						
Standard Deviation of Lateral Position[#]						
Mean (SD)	Placebo	1.09 (0.36)	1.07 (0.33)	1.06 (0.31)	1.10 (0.31)	1.10 (0.36)
	THC	1.15 (0.36)	1.22 (0.36)	1.27 (0.40)	1.23 (0.38)	1.22 (0.40)
Change from Time 1*	Placebo	--	.080	.080	.365	.365
	THC	--	.155	.001	.155	.155
Differences in Change*	THC v Placebo	--	.020	<.001	.094	.094
Time Out of Lane (seconds)[#]						
Mean (SD)	Placebo	2.46 (3.35)	2.24 (3.28)	2.22 (3.10)	2.39 (2.83)	2.86 (3.83)
	THC	2.51 (3.00)	3.07 (3.29)	3.31 (3.63)	2.66 (3.30)	2.69 (3.94)
Change from Time 1*	Placebo	--	.792	.792	.990	.990
	THC	--	.017	.003	.688	.840
Differences in Change*	THC v Placebo	--	.056	.022	.933	.947
Speed Deviation (mph)[#]						
Mean (SD)	Placebo	2.13 (1.81)	2.03 (1.31)	2.49 (1.70)	2.15 (1.89)	2.34 (1.82)
	THC	2.62 (1.96)	3.02 (1.88)	2.99 (1.97)	2.85 (1.97)	2.64 (1.94)
Change from Time 1*	Placebo	--	.794	.260	.794	.721
	THC	--	.007	.013	.172	.987
Differences in Change*	THC v Placebo	--	.469	.956	.721	.721
**mSuRT iPAD TASK**						
Number of Correct Hits						
Mean (SD)	Placebo	30.7 (1.80)	30.9 (1.71)	30.5 (1.62)	30.6 (2.03)	31.0 (1.44)
	THC	30.4 (1.80)	29.6 (3.39)	30.0 (2.21)	30.2 (2.16)	30.9 (1.48)
Change from Time 1*	Placebo	--	.620	.620	.665	.566
	THC	--	.011	.048	.323	.002
Differences in Change*	THC v Placebo	--	.036	.741	.848	.741
Distance from Target (pixels)[#]						
Mean (SD)	Placebo	19.9 (8.51)	20.3 (11.9)	22.2 (12.3)	21.8 (20.1)	17.5 (9.50)
	THC	20.8 (11.2)	27.0 (19.8)	26.3 (19.0)	22.3 (13.6)	18.0 (7.89)
Change from Time 1*	Placebo	--	.885	.465	.885	.030
	THC	--	<.001	<.001	.258	.001
Differences in Change*	THC v Placebo	--	.009	.433	.581	.814

SD = standard deviation; h = hour, min = minutes.

[#] Mean (SD) for raw scores are reported for interpretability. Analyses were conducted on the transformed values, as described in Methods.

* p-value; adjusted for multiple comparison using false discovery rate (FDR).

mSuRT = Modified Surrogate Reference Test

© 2022 Marcotte TD et al. *JAMA Psychiatry.*

eTable 1. Drive Subscores (continued)

		Time 1 (pre-smoke)	*Time 2 (30min)*	*Time 3 (1h 30min)*	*Time 4 (3h 30min)*	*Time 5 (4h 30min)*
**CAR FOLLOWING**						
Car following -- Coherence (correlation 0 -1)						
Mean (SD)	Placebo	0.76 (0.19)	0.79 (0.13)	0.77 (0.16)	0.80 (0.15)	0.75 (0.16)
	THC	0.75 (0.17)	0.68 (0.19)	0.66 (0.21)	0.74 (0.17)	0.73 (0.17)
Change from Time 1*	Placebo	--	.762	.780	.697	.762
	THC	--	.001	<.001	.753	.362
Differences in Change*	THC v Placebo	--	.020	.020	.261	.867
Car following – Delay in Response (seconds)						
Mean (SD)	Placebo	2.92 (1.15)	2.86 (1.05)	2.80 (0.99)	2.61 (1.04)	2.93 (1.05)
	THC	3.45 (1.24)	3.50 (1.10)	3.37 (1.16)	3.04 (1.13)	3.26 (1.16)
Change from Time 1*	Placebo	--	.939	.907	.267	.994
	THC	--	.729	.699	.004	.240
Differences in Change*	THC v Placebo	--	.816	.816	.816	.816
Car following – Distance from Lead Car						
Mean (SD)	Placebo	153 (41)	143 (36)	144 (34)	129 (33)	138 (34)
	THC	159 (38)	164 (39)	163 (37)	143 (36)	143 (37)
Change from Time 1*	Placebo	--	.030	.076	<.001	.003
	THC	--	.088	.286	<.001	<.001
Differences in Change*	THC v Placebo	--	.014	.078	.210	.845

SD = standard deviation; h = hour, min = minutes.
[#] Mean (SD) for raw scores are reported for interpretability. Analyses were conducted on the transformed values, as described in Methods.
* p-value; adjusted for multiple comparison using false discovery rate (FDR).

© 2022 Marcotte TD et al. *JAMA Psychiatry.*

eTable 2. Participant responses to self-report questionnaire after smoking, but prior to driving

2a. "How impaired are you to drive?" Self-rating from 0 (Not at all) to 100 (Extremely).

	Placebo	THC	Cliff's delta	p
30min	3.0 (0.0, 12.0)	30.0 (6.3, 73.8)	0.53	<.001
1h 30min	0.0 (0.0, 4.0)	14.5 (3.0, 51.3)	0.55	<.001
3h 30min	0.0 (0.0, 2.0)	3.0 (0.0, 15.0)	0.32	<.001
4h 30min	0.0 (0.0, 2.0)	0.0 (0.0, 6.3)	0.16	.026

Median (IQR)
Analyses of ranks; Cliff's delta compares THC to Placebo
p-values are adjusted for multiple comparisons using false discovery rate (FDR) method

2b. "Would you drive in your current state?". Percent reporting "yes."

	Placebo	THC	Odds Ratio	p
30min	85.7%	47.5%	0.15	<.001
1h 30min	93.7%	68.6%	0.15	.001
3h 30min	93.7%	89.9%	0.59	.512
4h 30min	92.1%	93.2%	1.17	.786

Odds ratio (THC vs Placebo)
p-values are adjusted for multiple comparisons using false discovery rate (FDR) method.

© 2022 Marcotte TD et al. *JAMA Psychiatry.*

eTable 3. Driving simulator self-assessment questionnaire by group for each time point after driving.

3a. How much did the study drug affect your driving? Self-rating from 0 (Not at all) to 100 (Extremely).

	Placebo	THC	Cliff's delta	p
30min	8.0 (0.0, 20.0)	37.0 (10.5, 70.0)	0.52	<.001
1h 30min	3.0 (0.0, 8.0)	19.0 (7.0, 57.5)	0.53	<.001
3h 30min	0.0 (0.0, 4.0)	6.0 (0.0, 20.0)	0.36	<.001
4h 30min	0.0 (0.0, 4.0)	3.0 (0.0, 22.0)	0.26	.001

Median (IQR)
Analyses of ranks; Cliff's delta compares THC to Placebo
p-values are adjusted for multiple comparisons using false discovery rate (FDR) method

3b. How well did you drive? Self-rating from 0 (Not at all well) to 100 (Extremely well).

	Placebo	THC	Cohen's d	p
30min	76.4 (15.8)	65.9 (23.1)	-0.79	.007
1h 30min	78.1 (17.1)	74.9 (18.7)	-0.23	.474
3h 30min	78.2 (19.4)	79.4 (18.4)	0.10	.663
4h 30min	83.4 (15.2)	80.3 (20.4)	-0.20	.474

Cohen's d compares THC to Placebo
p-values are adjusted for multiple comparisons using false discovery rate (FDR) method

© 2022 Marcotte TD et al. *JAMA Psychiatry.*

eTable 4. Correlation between blood THC levels and the Composite Driving Score at each timepoint.

Time after smoking	r (95% Confidence interval)	p
30min	0.025 (-0.152, 0.201)	.780
1h 30min	-0.021 (-0.207, 0.166)	.824
3h 30min	-0.017 (-0.210, 0.177)	.867
4h 30min	-0.130 (-0.317, 0.066)	.191

© 2022 Marcotte TD et al. *JAMA Psychiatry.*

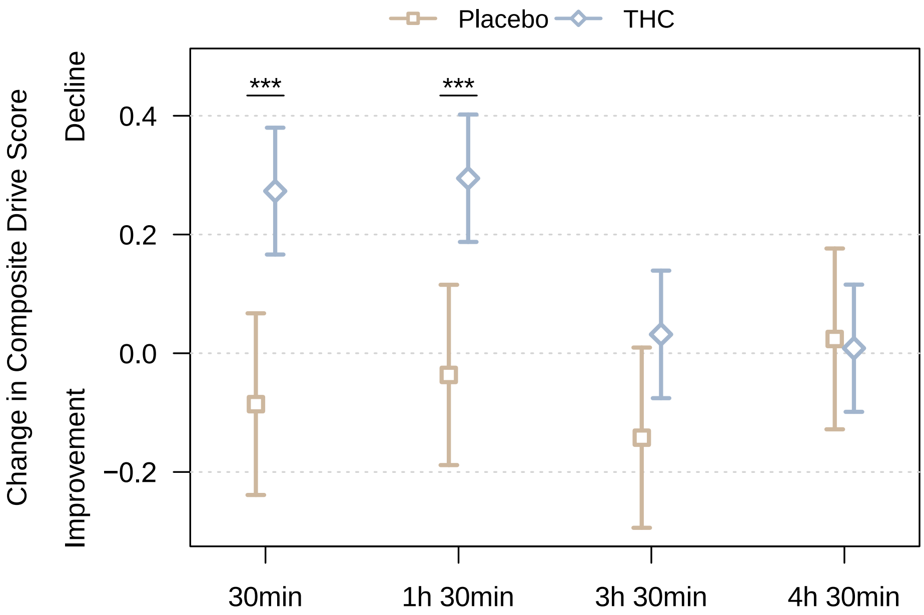

eFigure. Change in Composite Drive Score from baseline: THC groups combined. Values are means±95% CI. ***p<0.001

© 2022 Marcotte TD et al. *JAMA Psychiatry.*

eAppendix.

Driving simulations

Driving simulations: Driving simulations were presented on a STISIM M300WS-Console Driving Simulator System (Systems Technology, Inc; Hawthorne, CA) consisting of 3-screen, wide field-of-view monitors, steering wheel, and accelerator and brake pedals, and programmed using STISIM Drive v3.14[1]. The fully interactive simulation included routine and non-routine events throughout the drive. This included driving within residential, commercial and highway sections. During these drives participants encountered intersections, moving traffic, pedestrians, stop signs, and other challenges a driver may encounter on a road. Each simulation included intersections in which the participant would encounter the "yellow light dilemma", wherein individuals need to respond to a yellow light (timed to be consistent with California Law) and decide whether to stop or continue on, possibly risking running a red light; scenarios in which the participant is to merge with highway traffic, and quickly exit the roadway; make left turns in front of on-coming traffic, and other decision-making situations. Each drive also included a pre-determined crash avoidance scenario in which the participant drives down a visually complex roadway (moving cars, pedestrians) and encounters the sudden appearance of a pedestrian, or car pulling out, in the roadway. While all participants encountered the same scenarios, these were in part free drives in that the participant could adjust their speed, choose lane positions, etc. as desired. The simulations covered approximately 10.5 miles and took approximately 25 minutes to complete.

Importantly, within the context of this "normal" drive we included controlled scenarios that have previously been shown to be sensitive to acute cannabis use and other impairment-causing conditions at specified points in the simulation:

Modified Surrogate Reference Test (mSuRT). This is a divided attention task, modified from the Surrogate Reference Task[2], and developed in collaboration with colleagues at Brainbaseline®. Upon initiation of the task (a phone would ring), participants were asked to view an iPad, off to the side of the simulator monitors (Figure A). The iPad shows a pattern of random, hollow circles, with one of the circles being different (larger) than the others. The participant's task is to locate and touch the larger circle. The level of difficulty is varied by changing the ratio of the size of the distractor circles and target circles. Importantly, the participant is instructed to also maintain the appropriate speed (65 mph) and maintain the correct position in the center of the lane. In order for this to yield performance under a controlled condition (thus facilitating group analyses), no other traffic is on the road during this time. There are 16 trials each within the *easy* and *hard* versions. The next stimulus appears immediately after the participant's response. The secondary task (mSuRT) was presented at the same pre-determined location for all participants. They were expected to complete the task when it started. It takes approximately 60 seconds to complete the easy task, and 60-90 seconds for the hard task, depending upon how rapidly the participant responded to each stimulus.

This is a measure of performance under high cognitive load and controlled processing, in that participants must divide their attention among three stimuli (roadway, speedometer, and events in the periphery), and is reflective of the workload generated by a real task (e.g., a GPS system). While we considered using more face valid interfaces (such as an iPhone) and tasks (e.g., identifying musical tracks), this surrogate or structured task allow us to look at changes in attention in a more controlled fashion (not affected by familiarity with interface, reading speed, etc.).

© 2022 Marcotte TD et al. *JAMA Psychiatry.*

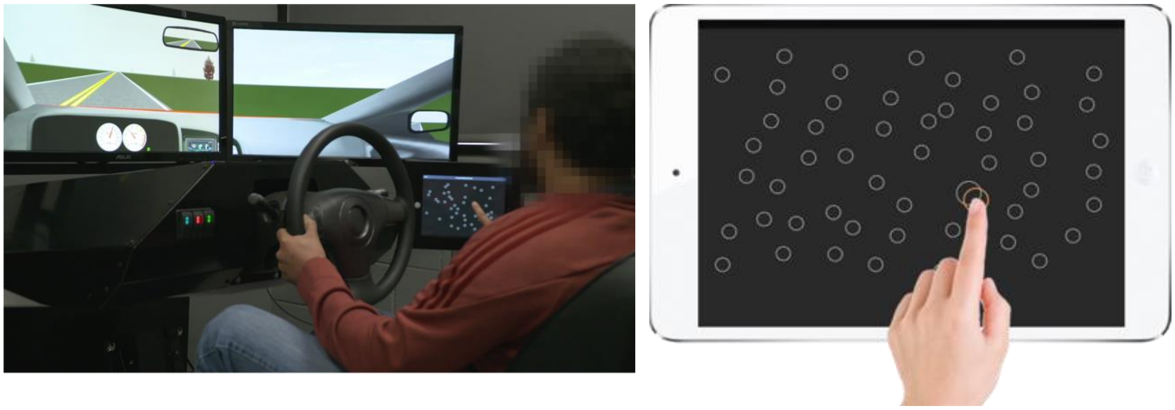

Figure A. Modified Surrogate Reference Task (mSuRT)

Car Following. Once during each simulation, at a specified location the participants were required to adjust their speed to a lead car that speeds up and slows down according to a sinusoidal wave. The primary outcome is the coherence between the participant and lead cars (a general correlation [0–1] of the participant's ability to accurately track the speed variations of the lead car). Time delay (or the reaction time to changes in the lead car's speed) and distance from the lead car were also variables of interest.

Note that individuals desiring greater details regarding the simulations may contact the lead investigator at tmarcotte@health.ucsd.edu.

Composite Drive Score. Driving simulators bring with them the ability to collect massive amounts of data. In some cases, even targeted scenarios have multiple outcomes of interest. This comes at a cost, though, in that it is not always clear regarding whether an individual, overall, evidenced a decline in driving performance. To address this, in addition to analyzing individual outcome variables we developed a Composite Drive Score that incorporates the key variables from the two scenarios above and combined them in a manner to create a single score. We then created a baseline anchor for performance based upon the performance of all 191 participants during their pre-smoking drive. All subsequent Composite Drive Scores used this as the basis for developing the score at each timepoint, thus facilitating analysis of change in performance from pre-treatment.

In order to accomplish this, z-scores were established based upon the pre-smoking simulator performance, using the mean and standard deviation on each score for all 191 participants. Z-scores for each participant were calculated by subtracting the group mean score from the participant's score and dividing that by the group standard deviation (so that, in the end, at the pre-smoke driving the Composite Drive Score for the entire sample had a mean z-score of 0, with a standard deviation of 1). Higher z-scores at each timepoint indicate worse performance (variables that went in the opposite direction were reflected in order to have all variables have the same direction). When examining the change in Composite Drive Score, a higher score indicates a decline in performance (e.g., Time 2 minus Time 1). The Composite Drive Score was comprised of the following variables: mSuRT task (SDLP, Speed Deviation, correct hits on SuRT) and Car Following (coherence).

The validity of SDLP and Car Following tasks in detecting declines in performance relating to cannabis and other substances has been widely reported[3-6; 7, 8; 9]. Developing a

© 2022 Marcotte TD et al. *JAMA Psychiatry.*

composite score, used frequently in other types of behavioral studies, overcomes one limitation in cannabis/driving studies noted in a recent comprehensive review– an emphasis on multiple dependent variables[10]. A similar approach has been used by others[11, 12], demonstrating sensitivity to cannabis consumption and aging. This is the first time incorporating these specific measures from this simulator into a composite score.

Determination of Impairment. Since there are no clear determinants of "impairment" for these experimental simulations, we established a cutpoint based upon the distribution of the change in CDS scores in the Placebo group. Based upon previous methodological work examining cutpoints for cognitive measures in relation to brain function[13, 14], which was subsequently further validated[15], we selected a cutpoint approximating the upper 15th percentile (based on higher CDS scores associated with worse performance). This resulted in 14.75% of the Placebo group being classified as impaired at the 30 min timepoint. Using this cutpoint, 45.60% of the THC group was classified as impaired at the same timepoint. While we could use a more conservative approach (e.g., a cutpoint of the 10th or 5th percentile), the previous work demonstrated that the 15th percentile cutpoint is optimal for detecting mild impairments.

Whole Blood Collection and THC Analyses

Venous blood was collected from an indwelling intravenous catheter in the arm into vacutainer tubes containing sodium fluoride (NaF) and potassium oxalate. Following collection, whole blood was transferred to Nunc cryovials (Wheaton, Millville, NJ) and stored at -20 °C for a maximum of three months prior to analysis. Samples were prepared and analyzed by isotope-dilution LC-MS/MS using validated, published methods[16]. Sample collection time was calculated based on time elapsed from the start of smoking.

Cannabis Use History ("Use Intensity")

In order to estimate recent cannabis use history (THC exposure), participants completed a structured, timeline follow-back interview regarding their frequency of cannabis use and estimated quantity of use in the past 6 months. This was used to establish a measure of use intensity. While days of use is informative, it does not account for the amount that a person may use each day. Of particular interest are the effects of possible tolerance in those who use the most or the least. Thus, based upon the distribution of estimated THC exposure (quantity x frequency) in the past 6 months for all 191 participants, we separated the group based upon quartiles – the highest quartile (≥ 132 grams; n = 46), the middle two quartiles (17-132 grams; n = 92), and the lowest quartile (≤ 17 grams; n = 46).

Randomization/Blinding

Treatment groups were assigned using permuted blocks randomization with stratification by prior cannabis exposure (frequent user [≥4 times per week] versus occasional user [<4 times per week]). The allocation schedule was kept in the UCSD Research Pharmacy, which prepared the cannabis material, and the schedule was concealed from other study personnel. Participants and assessors were blinded to group assignments.

Statistical analysis

All analyses were performed using R v.4.0.3 via RStudio v.1.3.1093[17]. Demographic and other relevant characteristics were compared between groups using ANOVA, Kruskal-Wallis test, chi-

© 2022 Marcotte TD et al. *JAMA Psychiatry.*

square test, and Fisher's exact test as appropriate. Two-group comparisons were carried out using t-test (or Wilcoxon), chi-square test, or Fisher's exact test as dictated by the procedures' assumptions.

The study outcomes were analyzed using methods for longitudinal data and repeated measures as described in the Statistical Analysis section of the manuscript. For all models, three terms were included: treatment (Placebo, THC), time (5 time points), and the treatment-time interaction. A significant interaction p-value (<0.05) indicates that the changes in the outcome over time are statistically different between treatments, thus signifying treatment effect. To meet the assumption of normality, some continuous variables were standardized. To meet the assumptions for Poisson distribution, some discrete variables were reverse-coded (by subtracting the variable values from its possible maximum).

The effect size for continuous outcomes was estimated by Cohen's d (standardized difference) calculated by dividing the model coefficients with residual standard error, or by Cliff's delta[18]. Confidence intervals (CI) at 95% level were calculated for all effect sizes. Confidence intervals reported with p-values that were adjusted for multiple testing were also corrected using false discovery (FDR) method.

Power/sample size. In a previous study using a single monitor and less challenging divided attention task (stimuli would appear on the screen itself) we found that participants who smoked cannabis cigarettes with 4% THC evidenced effect sizes between 0.36 and 0.47 when comparing changes in SDLP between placebo and active THC at 2 to 3 hours post-smoking[19]. For power calculations for this study, it was assumed that the placebo group will show minimal changes in CDS over time and that the 13.4% THC group will show a worsening in CDS immediately after smoking cannabis with a gradual return to expected CDS levels afterwards. Cohen's d was used as an estimate for the effect size for measuring the difference in changes in CDS from baseline (pre-cannabis) to the time point with the assumed largest differences between the two groups. Under these assumptions, power for finding a significant difference in changes in CDS between the 13.4% THC group (n = 60) and the placebo (n = 60) was estimated using 1000 simulations, which showed 80% power to detect Cohen's d=0.33 or larger with significance level α=0.05.

Adverse events

There were no serious adverse events recorded in the study. A total of twenty-four participants (12% out of the 199 enrolled) reported experiencing one to five adverse events for a total of 46 events, including 44 mild (grade 1) and 2 moderate (grade 2). During the screening visit, 6 adverse events, consisting of dizziness (1), nausea (3), and vomiting (2), were reported by 4 participants. Since smoking did not occur, these were associated with driving simulator-induced motion sickness.

At the primary study visit, 22 participants (Placebo: n=2, 5.9% THC: n=15, 13.4% THC: n=5; Fisher's Exact test p=.002) reported 40 adverse events (Placebo: 2, 5.9% THC: 30, 13.4% THC: 8). Most common symptoms were abnormal heart rate (13), dizziness (6), changes in blood pressure (4 decreased, 2 increased) and nausea (3). Other reported symptoms included anxiety (2), discomfort (2), sweats (2), and one of each for cough, fainting, fever, headache, numbness in the arm, and swollen arm. Other than one participant who withdrew due to anxiety, all AEs resolved and participants continued with their visit.

eReferences

1. Rosenthal TJ. *STISIM Drive*. Systems Technology, Inc.; 2017.
2. Ostlund J, Nilsson L, Tomros J, Forsman A. *Calibration tasks for methods which assess demand due to the use of invehicle systems*. 2006.
3. Bosker WM, Kuypers KP, Theunissen EL, et al. Medicinal Delta(9) -tetrahydrocannabinol (dronabinol) impairs on-the-road driving performance of occasional and heavy cannabis users but is not detected in Standard Field Sobriety Tests. *Addiction*. Oct 2012;107(10):1837-44. doi:10.1111/j.1360-0443.2012.03928.x
4. Hartman RL, Brown TL, Milavetz G, et al. Cannabis effects on driving lateral control with and without alcohol. *Drug Alcohol Depend*. Sep 1 2015;154:25-37. doi:10.1016/j.drugalcdep.2015.06.015
5. Arkell TR, Lintzeris N, Kevin RC, et al. Cannabidiol (CBD) content in vaporized cannabis does not prevent tetrahydrocannabinol (THC)-induced impairment of driving and cognition. *Psychopharmacology*. 2019;236(9):2713-2724.
6. Ramaekers JG, Robbe HW, O'Hanlon JF. Marijuana, alcohol and actual driving performance. *Hum Psychopharmacol*. Oct 2000;15(7):551-558. doi:10.1002/1099-1077(200010)15:7<551::AID-HUP236>3.0.CO;2-P
7. Lenne MG, Dietze PM, Triggs TJ, Walmsley S, Murphy B, Redman JR. The effects of cannabis and alcohol on simulated arterial driving: Influences of driving experience and task demand. *Accid Anal Prev*. May 2010;42(3):859-66. doi:10.1016/j.aap.2009.04.021
8. Brown TL, Richard C, Meghdadi A, et al. EEG biomarkers acquired during a short, straight-line simulated drive to predict impairment from cannabis intoxication. *Traffic Inj Prev*. Sep 25 2020:1-5. doi:10.1080/15389588.2020.1814957
9. Hartman RL, Brown TL, Milavetz G, et al. Cannabis effects on driving longitudinal control with and without alcohol. *J Appl Toxicol*. Nov 2016;36(11):1418-29. doi:10.1002/jat.3295
10. Alvarez L, Colonna R, Kim S, et al. Young and under the influence: A systematic literature review of the impact of cannabis on the driving performance of youth. *Accid Anal Prev*. Mar 2021;151:105961. doi:10.1016/j.aap.2020.105961
11. Ortiz-Peregrina S, Ortiz C, Castro-Torres JJ, Jimenez JR, Anera RG. Effects of Smoking Cannabis on Visual Function and Driving Performance. A Driving-Simulator Based Study. *Int J Environ Res Public Health*. Dec 3 2020;17(23)doi:10.3390/ijerph17239033
12. Wood JM. Age and visual impairment decrease driving performance as measured on a closed-road circuit. *Hum Factors*. Fall 2002;44(3):482-94. doi:10.1518/0018720024497664
13. Tulsky DS, Saklofske DH, Chelune GJ, et al, eds. *Clinical interpretation of the WAIS-III and WMS-III*. Academic Press; 2003.
14. Heaton RK, Miller SW, Taylor MJ, Grant I. *Revised Comprehensive norms for an expanded Halstead-Reitan Battery: Demographically adjusted neuropsychological norms for African American and Caucasian adults*. Psychological Assessment Resources, Inc.; 2004.
15. Campbell LM, Fennema-Notestine C, Saloner R, et al. Use of Neuroimaging to Inform Optimal Neurocognitive Criteria for Detecting HIV-Associated Brain Abnormalities. *J Int Neuropsychol Soc*. Feb 2020;26(2):147-162. doi:10.1017/S1355617719000985
16. Hubbard JA, Smith BE, Sobolesky PM, et al. Validation of a liquid chromatography tandem mass spectrometry (LC-MS/MS) method to detect cannabinoids in whole blood and breath. *Clin Chem Lab Med*. Apr 28 2020;58(5):673-681. doi:10.1515/cclm-2019-0600
17. R Core Team. *R: A language and environment for statistical computing*. 2020.
18. Cliff N. Dominance statistics: Ordinal analyses to answer ordinal questions. *Psychological Bulletin*. 1993;114(3):494-509.

© 2022 Marcotte TD et al. *JAMA Psychiatry.*

19. Marcotte TD, Wolfson T, Meyer R, et al. Driving performance at clinically effective cannabis doses for the treatment of MS spasticity. *Journal of the International Neuropsychological Society*. 2015;21(1):123.

© 2022 Marcotte TD et al. *JAMA Psychiatry.*
