# Supplementary material for: Driving Performance and Cannabis Users’ Perception of Safety: A Randomized Clinical Trial
Source: JAMA Psychiatry. 2022 Jan 26;79(3):1–9. doi: 10.1001/jamapsychiatry.2021.4037 (PMC8792796; doi:10.1001/jamapsychiatry.2021.4037)
Supplement: Supplement 3. — Data Sharing Statement [file jamapsychiatry-e214037-s003.pdf]

Data Sharing Statement

Marcotte. Driving Performance and Cannabis Users' Perception of Safety. *JAMA Psychiatry*. Published January 26, 2022. doi:10.1001/jamapsychiatry.2021.4037

Data
Data available: Yes
Data types: Deidentified participant data, Data dictionary
How to access data: Data can be requested from cmcr@ucsd.edu
When available: With publication

Supporting Documents
Document types: None

Additional Information
Who can access the data: Approved researchers
Types of analyses: Meta-analysis
Mechanisms of data availability: With a signed data use agreement
